# Supplementary figures and images for: The Genomic landscape of short tandem repeats across multiple ancestries
Source: PLoS One. 2023 Jan 26;18(1):e0279430. doi: 10.1371/journal.pone.0279430 (PMC9879404; doi:10.1371/journal.pone.0279430)

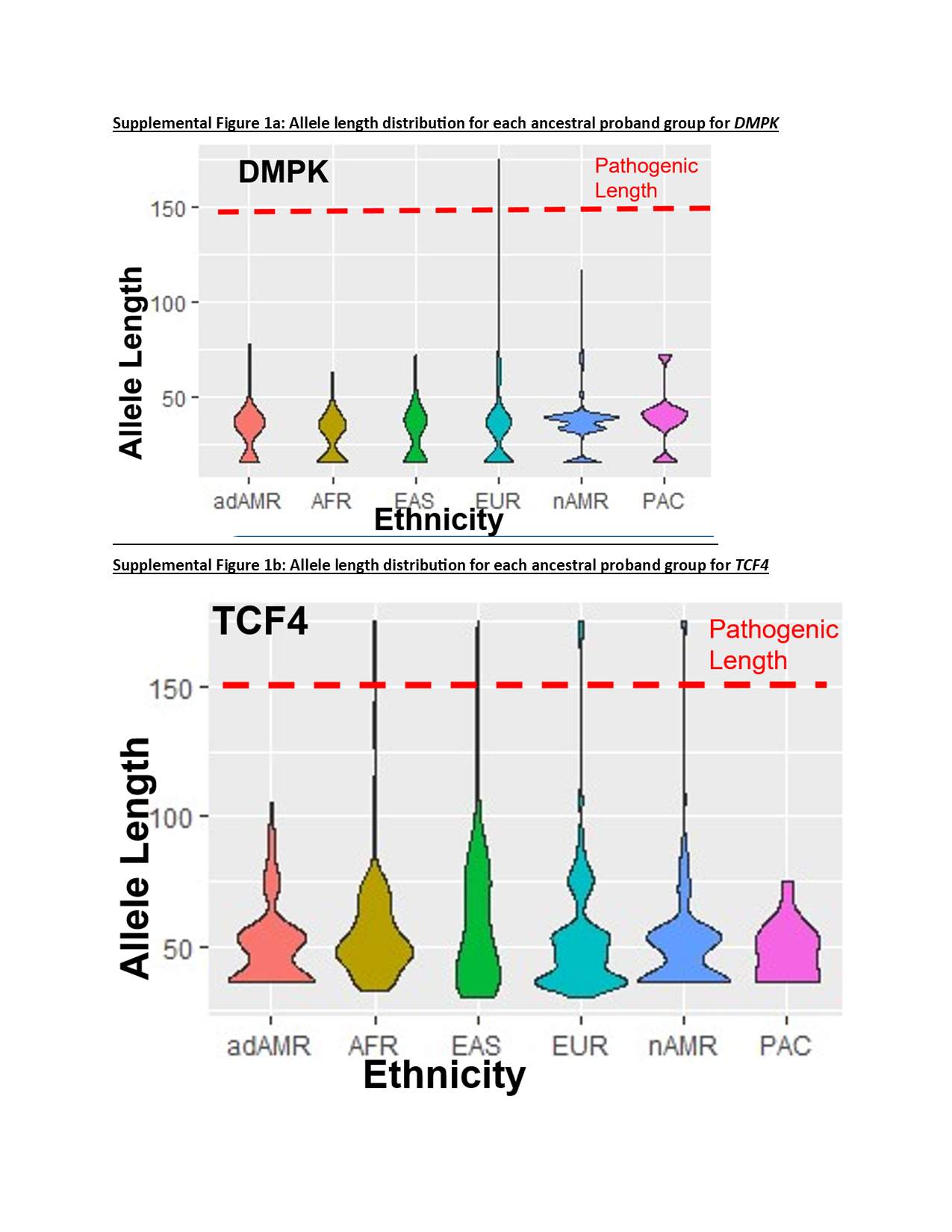

Supplement: S1 Fig — A: DMPK B: TCF4. (TIF) [file pone.0279430.s001.tif]

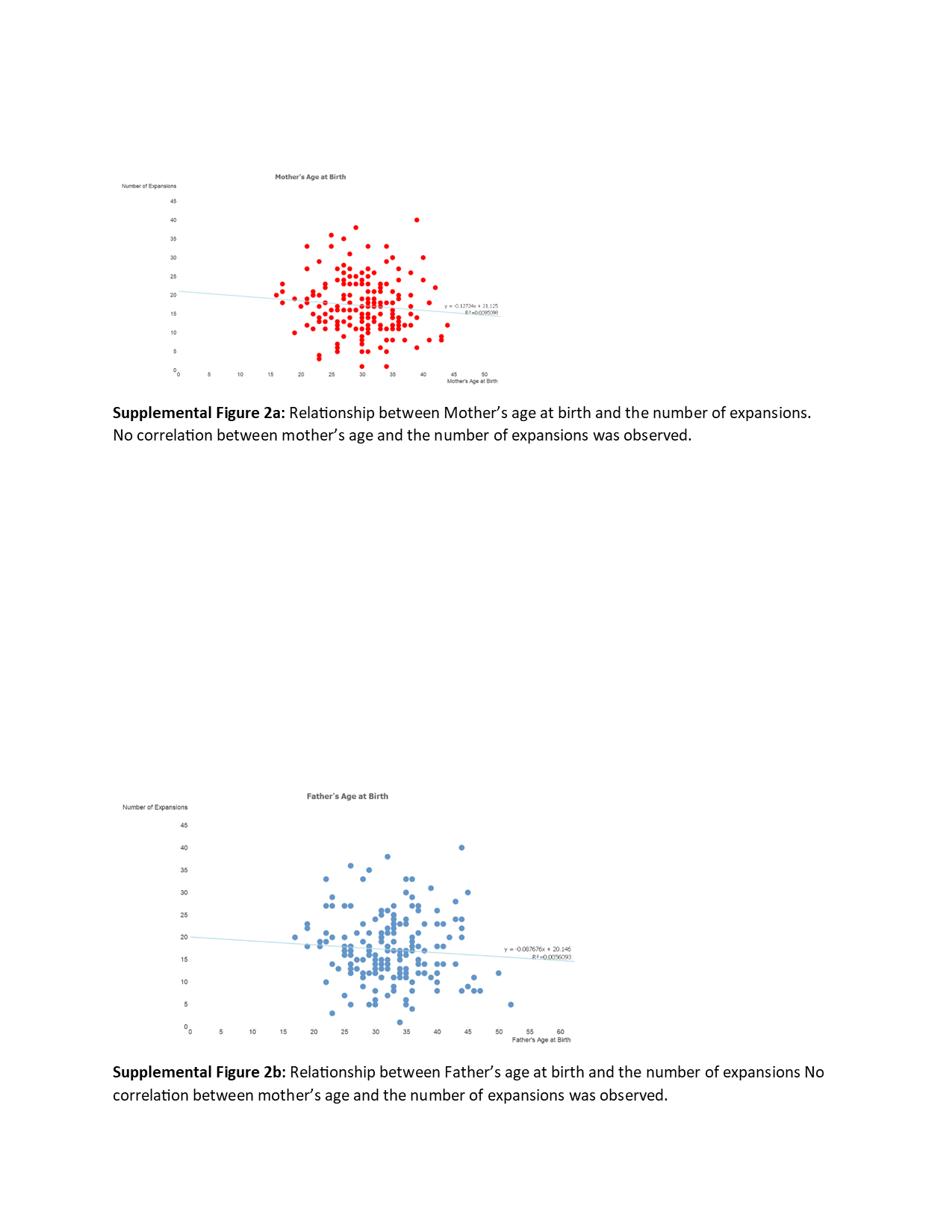

Supplement: S2 Fig — A. Correlation between Mother’s age and number of expansions. B. Correlation between Father’s age and number of expansions. (TIF) [file pone.0279430.s002.tif]
