# Supplementary material for: The Genomic landscape of short tandem repeats across multiple ancestries
Source: PLoS One. 2023 Jan 26;18(1):e0279430. doi: 10.1371/journal.pone.0279430 (PMC9879404; doi:10.1371/journal.pone.0279430)
Supplement: S1 Table — (DOCX) [file pone.0279430.s003.docx]

[Supplemental Table 1 a -e]

**A:**

| **Chromosome** | **HG 19 Position** | **EUR Avg Allele Length (bp)** | **Admixed American Avg Allele Length (bp)** | **Abs Z Score** | **Percent Difference (compared to EUR)** | **Gene** |
| --- | --- | --- | --- | --- | --- | --- |
| X | 72059995 | 21.40 | 46.78 | 7.34 | 218.58% | N/A |
| 12 | 114053434 | 7.21 | 14.54 | 6.77 | 201.63% | N/A |
| X | 86137693 | 11.76 | 23.55 | 11.05 | 200.29% | N/A |
| 3 | 112188455 | 11.08 | 20.48 | 8.30 | 184.80% | BTLA |
| 16 | 87053679 | 4.59 | 8.45 | 9.94 | 184.09% | N/A |
| 9 | 12182163 | 8.35 | 15.20 | 12.56 | 182.14% | N/A |
| 17 | 73461737 | 10.65 | 19.39 | 16.93 | 182.06% | *KIAA0195* |
| 17 | 4972472 | 16.47 | 29.81 | 5.66 | 181.05% | N/A |
| 19 | 59035273 | 3.73 | 6.59 | 10.42 | 176.92% | N/A |
| 9 | 12182185 | 8.79 | 15.11 | 11.88 | 172.04% | N/A |
| 9 | 133383220 | 10.94 | 18.63 | 10.89 | 170.33% | N/A |
| 13 | 34435040 | 5.87 | 9.98 | 12.74 | 169.97% | RFC3 |
| X | 871016 | 36.87 | 61.46 | 7.14 | 166.69% | N/A |
| 7 | 112103909 | 16.10 | 26.82 | 9.73 | 166.60% | *IFRD1* |
| 17 | 7475156 | 17.96 | 29.45 | 6.69 | 164.00% | *SENP3* |
| X | 152356423 | 6.41 | 10.32 | 11.13 | 160.92% | N/A |
| 6 | 114184967 | 18.05 | 28.60 | 11.87 | 158.47% | *MARCKS* |
| 4 | 151227792 | 3.85 | 6.08 | 13.15 | 157.97% | *LRBA* |
| X | 38068240 | 7.34 | 11.56 | 14.74 | 157.52% | *SRPX* |
| 10 | 115299575 | 24.71 | 38.81 | 10.31 | 157.08% | N/A |

**B:**

| **Chromosome** | **HG19 Position** | **EUR Avg Allele Length (bp)** | **PAC Avg Allele Length (bp)** | **Abs Z Score** | **Percent Difference (compared to EUR)** | **Gene** |
| --- | --- | --- | --- | --- | --- | --- |
| 14 | 78886305 | 7.15 | 20.58 | 6.09 | 288% | *NRXN3* |
| X | 68641258 | 2.59 | 6.69 | 9.32 | 258% | N/A |
| 17 | 63966940 | 20.75 | 52.11 | 5.43 | 251% | *CEP112* |
| 2 | 39828643 | 6.64 | 15.63 | 5.76 | 236% | *AC007246N/A3* |
| 2 | 57896350 | 12.61 | 29.58 | 6.22 | 235% | N/A |
| 7 | 20319311 | 30.22 | 70.53 | 5.48 | 233% | N/A |
| 14 | 106042736 | 4.48 | 10.42 | 9.97 | 232% | *TMEM121*(dist-46198) |
| 4 | 9616936 | 8.03 | 18.63 | 5.85 | 232% | N/A |
| 4 | 9573183 | 4.42 | 9.89 | 8.22 | 224% | N/A |
| 9 | 12182163 | 8.35 | 18.00 | 8.20 | 216% | N/A |
| 9 | 12182185 | 8.79 | 18.25 | 9.14 | 208% | N/A |
| 12 | 19487764 | 16.66 | 34.53 | 39.79 | 207% | *PLEKHA5* |
| 2 | 13845116 | 7.30 | 15.05 | 7.96 | 206% | N/A |
| 14 | 60441785 | 6.78 | 13.89 | 6.32 | 205% | *LRRC9* |
| 14 | 60441753 | 6.62 | 13.47 | 6.19 | 203% | *LRRC9* |
| 7 | 112103909 | 16.10 | 32.56 | 6.51 | 202% | *IFRD1* |
| 14 | 106047470 | 5.26 | 10.58 | 8.45 | 201% | *TMEM121*(dist-50932) |
| 9 | 133383220 | 10.94 | 21.81 | 6.76 | 199% | N/A |
| 1 | 6587680 | 8.48 | 16.05 | 7.02 | 189% | *NOL9* |
| 13 | 34435040 | 5.87 | 11.11 | 7.12 | 189% | *RFC3* |

**C:**

| **Chromosome** | **HG19 Position** | **EUR Avg Allele Length (bp)** | **Non-Admixed American Avg Allele Length (bp)** | **Abs Z Score** | **Percent Difference (compared to EUR)** | **Gene** |
| --- | --- | --- | --- | --- | --- | --- |
| 3 | 112188455 | 11.08 | 26.65 | 14.85 | 240% | *BTLA* |
| X | 86137693 | 11.76 | 26.38 | 14.20 | 224% | N/A |
| 9 | 12182163 | 8.35 | 17.24 | 19.23 | 207% | N/A |
| 6 | 67684311 | 6.86 | 13.71 | 6.13 | 200% | N/A |
| 10 | 115299575 | 24.71 | 49.21 | 20.93 | 199% | N/A |
| X | 72059995 | 21.40 | 42.57 | 7.55 | 199% | N/A |
| 9 | 133383220 | 10.94 | 21.75 | 19.44 | 199% | N/A |
| 16 | 87053679 | 4.59 | 9.02 | 12.71 | 197% | N/A |
| 13 | 34435040 | 5.87 | 11.53 | 21.81 | 196% | RFC3 |
| 9 | 12182185 | 8.79 | 17.18 | 18.64 | 196% | N/A |
| 13 | 30363008 | 2.77 | 5.40 | 8.33 | 195% | UBL3 |
| X | 871016 | 36.87 | 71.20 | 11.89 | 193% | N/A |
| 9 | 41832389 | 10.76 | 20.60 | 14.49 | 191% | N/A |
| 7 | 1321608 | 5.93 | 11.33 | 13.80 | 191% | N/A |
| 17 | 73461737 | 10.65 | 20.21 | 21.46 | 190% | KIAA0195 |
| 5 | 30518243 | 7.53 | 14.25 | 6.19 | 189% | N/A |
| 12 | 114053434 | 7.21 | 13.46 | 6.65 | 187% | N/A |
| 11 | 67115105 | 16.23 | 30.25 | 17.23 | 186% | LOC100130987 |
| 19 | 59035273 | 3.73 | 6.93 | 12.87 | 186% | N/A |
| 8 | 9119983 | 15.53 | 28.38 | 12.03 | 183% | N/A |

**D:**

| **Chromosome** | **HG19 Position** | **EUR Avg Allele Length (bp)** | **EAS Avg Allele Length (bp)** | **Abs Z Score** | **Percent Difference (compared to EUR)** | **Gene** |
| --- | --- | --- | --- | --- | --- | --- |
| 14 | 78886305 | 7.15 | 20.18 | 13.61 | 282% | *NRXN3* |
| 5 | 30518243 | 7.53 | 20.15 | 7.59 | 268% | N/A |
| X | 72059995 | 21.40 | 56.38 | 7.61 | 263% | N/A |
| 8 | 30000883 | 5.96 | 14.66 | 8.13 | 246% | *MIR548O2* |
| 4 | 9616936 | 8.03 | 19.50 | 15.25 | 243% | N/A |
| 1 | 54070575 | 2.44 | 5.67 | 9.64 | 233% | *GLIS1* |
| 5 | 53206464 | 12.92 | 29.07 | 12.93 | 225% | *ARL15* |
| 7 | 112103909 | 16.10 | 35.65 | 20.88 | 221% | *IFRD1* |
| 9 | 12182163 | 8.35 | 18.17 | 19.92 | 218% | N/A |
| 7 | 131352014 | 8.93 | 19.38 | 12.54 | 217% | N/A |
| 13 | 89814157 | 4.25 | 9.10 | 9.81 | 214% | N/A |
| X | 871016 | 36.87 | 77.85 | 9.79 | 211% | N/A |
| X | 68641258 | 2.59 | 5.44 | 13.13 | 210% | N/A |
| 6 | 67684311 | 6.86 | 14.42 | 5.49 | 210% | N/A |
| 9 | 12182185 | 8.79 | 18.18 | 19.14 | 207% | N/A |
| 13 | 63318290 | 34.76 | 71.73 | 10.54 | 206% | N/A |
| 4 | 9573183 | 4.42 | 9.12 | 14.78 | 206% | N/A |
| 2 | 13845116 | 7.30 | 15.03 | 17.42 | 206% | N/A |
| 8 | 132332242 | 13.90 | 28.30 | 9.80 | 204% | N/A |
| 14 | 106042736 | 4.48 | 9.05 | 14.92 | 202% | *TMEM121*(dist-46198) |

**E:**

| **Chromosome** | **HG19 Position** | **EUR Avg Allele Length (bp)** | **AFR Avg Allele Length (bp)** | **Abs Z Score** | **Percent Difference (compared to EUR)** | **Gene** |
| --- | --- | --- | --- | --- | --- | --- |
| 18 | 50168385 | 3.68 | 13.70 | 13.11 | 372% | *DCC* |
| 3 | 128925896 | 3.94 | 13.29 | 30.32 | 337% | N/A |
| 1 | 54070575 | 2.44 | 7.71 | 17.74 | 316% | *GLIS1* |
| 4 | 79109434 | 5.67 | 17.91 | 11.69 | 316% | *FRAS1* |
| 10 | 46710300 | 4.47 | 14.08 | 12.62 | 315% | N/A |
| 12 | 21803040 | 6.02 | 18.64 | 10.69 | 309% | *LDHB* |
| 1 | 101033552 | 7.89 | 24.35 | 15.54 | 309% | N/A |
| 4 | 86026772 | 9.38 | 28.56 | 27.97 | 304% | N/A |
| 1 | 248118248 | 4.47 | 13.60 | 29.31 | 304% | *OR2L13* |
| 3 | 112188455 | 11.08 | 33.32 | 23.19 | 301% | *BTLA* |
| 18 | 3551158 | 2.60 | 7.69 | 9.28 | 296% | *DLGAP1* |
| 9 | 76160643 | 8.28 | 24.42 | 15.42 | 295% | N/A |
| 2 | 21027058 | 3.36 | 9.75 | 27.02 | 290% | N/A |
| 16 | 29766639 | 6.15 | 17.69 | 37.65 | 288% | N/A |
| 16 | 85728017 | 3.46 | 9.82 | 14.74 | 284% | N/A |
| 4 | 64534252 | 3.53 | 9.86 | 19.90 | 280% | N/A |
| 17 | 58834690 | 11.50 | 31.32 | 37.72 | 272% | *BCAS3* |
| 15 | 48683912 | 5.53 | 14.99 | 11.88 | 271% | N/A |
| X | 124409230 | 8.82 | 23.74 | 6.11 | 269% | N/A |
| X | 68641258 | 2.59 | 6.86 | 30.84 | 265% | N/A |

**Supplemental Table 1 a-e. STR Length Variability Between Ancestries**: **A:** EUR vs Admixed AMR **B:** EUR vs PAC **C:** EUR vs Nonadmixed AMR **D:** EUR vs EAS **E:** EUR vs AFR
