# Supplementary material for: The Genomic landscape of short tandem repeats across multiple ancestries
Source: PLoS One. 2023 Jan 26;18(1):e0279430. doi: 10.1371/journal.pone.0279430 (PMC9879404; doi:10.1371/journal.pone.0279430)
Supplement: S2 Table — * This position corresponds to two different genes. (DOCX) [file pone.0279430.s004.docx]

[Supplemental Table 2]

| **Chromosome** | **Position** | **Gene** | **Population CohorotAverage** | **AFR Avg** | **Admixed AMR Avg** | **Non Admixed AMR Avg** | **EAS Avg** | **EUR Avg** | **PAC Avg** |
| --- | --- | --- | --- | --- | --- | --- | --- | --- | --- |
| 2 | 191745599 | *GLS* | 39.42 | 39.11 | 38.96 | 38.70 | 36.08 | 39.77 | 36.19 |
| 3 | 63898361 | *ATXN7* | 31.29 | 30.78 | 31.61 | 31.27 | 31.07 | 31.33 | 32.50 |
| 3 | 128891501 | *CNBP* | 36.14 | 34.86 | 36.59 | 35.94 | 32.92 | 36.67 | 33.75 |
| 4 | 3076604 | *HTT* | 55.04 | 53.76 | 55.83 | 54.98 | 53.25 | 55.26 | 53.06 |
| 4 | 39350045 | *MIR1273H,RFC1** | 64.18 | 65.04 | 60.43 | 58.96 | 53.93 | 65.85 | 59.40 |
| 4 | 41748008 | *PHOX2B* | 12.06 | 12.00 | 12.00 | 12.01 | 12.04 | 12.07 | 12.00 |
| 5 | 146258291 | *PPP2R2B* | 35.22 | 35.12 | 36.80 | 36.24 | 38.13 | 34.66 | 36.86 |
| 11 | 119077000 | *CBL* | 34.65 | 32.72 | 33.81 | 33.68 | 35.49 | 34.98 | 35.00 |
| 12 | 7045892 | *ATN1* | 41.73 | 38.96 | 42.61 | 41.38 | 43.63 | 41.80 | 45.50 |
| 12 | 50898785 | *DIP2B* | 28.03 | 24.77 | 25.33 | 23.73 | 21.48 | 29.67 | 23.19 |
| 14 | 92537355 | *ATXN3* | 43.73 | 49.97 | 41.48 | 41.39 | 43.23 | 43.29 | 44.63 |
| 16 | 87637894 | *JPH3* | 44.13 | 44.83 | 43.48 | 43.79 | 42.77 | 44.30 | 43.06 |
| 18 | 53253385 | *TCF4* | 58.22 | 56.75 | 53.61 | 57.07 | 60.33 | 59.09 | 55.00 |
| 19 | 13318673 | *CACNA1A* | 34.42 | 34.11 | 34.69 | 34.37 | 34.40 | 34.35 | 32.00 |
| 19 | 46273463 | *DMPK* | 36.57 | 34.07 | 35.00 | 38.50 | 37.05 | 36.60 | 40.38 |
| 20 | 2633379 | *NOP56* | 27.76 | 27.44 | 27.52 | 26.88 | 28.02 | 27.71 | 28.38 |
| 22 | 46191235 | *ATXN10* | 70.05 | 67.58 | 70.52 | 70.46 | 71.86 | 69.99 | 72.19 |
| X | 66765149 | *AR* | 16.66 | 14.04 | 16.37 | 18.92 | 20.71 | 16.33 | 18.36 |

**Supplemental Table 2**. Pathogenic STR Length by Ancestry: This table shows the most common pathogenic STR loci in the proband cohort. * This position corresponds to two different genes.

**AFR=African;AMR=American;EAS=EastAsian;PAC=PacificIslander;**
