# Supplementary material for: The Genomic landscape of short tandem repeats across multiple ancestries
Source: PLoS One. 2023 Jan 26;18(1):e0279430. doi: 10.1371/journal.pone.0279430 (PMC9879404; doi:10.1371/journal.pone.0279430)
Supplement: S1 Document — (DOCX) [file pone.0279430.s006.docx]

**S1 Document. GangSTR and DumpSTR scripts used on all files**.

- GangSTR Script Used for All VCFs
  - GangSTR-2.4/bin/GangSTR --ref ~/human_g1k_v37_decoy.fasta -- regions ~/hg19_ver10.sorted.bed.nochr --out <Output.vcf> --bam <File.bam>
- DumpSTR Filters
  - DumpSTR --vcf <File.vcf> --out <File.DumpSTRFiltered.vcf --vcftype gangstr --g --gangstr-min-call-DP 50 --gangstr-max-call-DP 1000 --gangstr-min-call-Q 0.90
