# Supplementary material for: The Genomic landscape of short tandem repeats across multiple ancestries
Source: PLoS One. 2023 Jan 26;18(1):e0279430. doi: 10.1371/journal.pone.0279430 (PMC9879404; doi:10.1371/journal.pone.0279430)
Supplement: Attachment — Submitted filename: Response to Reviewers.docx [file pone.0279430.s007.docx]

Response to Reviewers

**Journal Requirements**

- Please ensure that your manuscript meets PLOS ONE's style requirements, including those for file naming
  - Our manuscript was edited to fit with PLOS ONE’s style and file naming requirements
- Please include your full ethics statement in the ‘Methods’ section of your manuscript file.
  - Our ethics statement can now be found in the ‘Method’ section of our manuscript file.
- Please remove any funding-related text from the manuscript and let us know how you would like to update your Funding Statement
  - An amended funding statement can now be found in the cover letter for this revised submission
- We note that you have stated that you will provide repository information for your data at acceptance. Should your manuscript be accepted for publication, we will hold it until you provide the relevant accession numbers or DOIs necessary to access your data.
  - The information repository containing all data used in this study can be found in DNA Nexus and can be accessed via the following url: https://platform.dnanexus.com/projects/G6vJyPj0BYFv2KBJ4f55jkgf/data/
- Please include captions for your Supporting Information files at the end of your manuscript, and update any in-text citations to match accordingly.
  - Supporting information captions are now included at the end of the manuscript

**Review Comments**

- The analysis in the manuscript is too simple comparing to the huge data they used. In the first part, they can try to find some AIM-STRs from the 850,000 STRs on the 6 groups. They can also perform some population genetic analysis based on the STR data (e.g. Principal component analysis).
  - Although we agree it is an intriguing idea to conduct PCA on the STR data and we considered using some machine learning models to see if we could differentiate ethnicities based off of STR only we ultimately decided that that work is beyond the scope of this publication.
- In the second part, the authors examined probands to identify STR expansions at known pathogenic loci. However, the results presented is not very clear. I suggest authors to summarize the results to a figure/table or both.
  - We agree that there was some ambiguity in the results section dedicated to presenting the data on STR expansions in known pathogenic loci of probands. In response, that section of the manuscript has been modified to give more clarity. Additionally, **Table 3**, which summarizes the data contained in this section has been changed in order to better present the data gathered.
- Line 151. Which figure is quoted here is not clearly described.
  - The supplemental figure which is referenced in this line has been properly labelled.
- It is unclear the raw data is obtained from publicly available database or sequenced in the authors’ lab.
  - An additional statement is now included in the ‘Methods’ section which clarifies that the raw data was sequenced by Rady Children’s Institute for Genomic Medicine
- The data availability to the pubic should be provided.
  - The information repository containing all data used in this study can be found in DNA Nexus and can be accessed via the following url: https://platform.dnanexus.com/projects/G6vJyPj0BYFv2KBJ4f55jkgf/data/
- The tables provided is not in a standard format and the data is not summarized as a scientific paper.
  - Changes have been made throughout the paper to better present our findings. Tables have also been modified to fit the standard format.
- My concern is about M&M section... where authors just have written the names of software but didnt provide any script how these will work with this type of data... I would suggest to give scripts as ESM which will not only make MS more sound but attract more citations.
  - We acknowledge that it would be greatly beneficial if we provided the scripts used for GangSTR and the DumpSTR filters. To address this, we have included a supplemental document containing all pertinent scripts.
