# Supplementary material for: The Genomic landscape of short tandem repeats across multiple ancestries
Source: PLoS One. 2023 Jan 26;18(1):e0279430. doi: 10.1371/journal.pone.0279430 (PMC9879404; doi:10.1371/journal.pone.0279430)
Supplement: Attachment — Submitted filename: Response to Reviewers.docx [file pone.0279430.s008.docx]

Response to Reviewers

**Journal Requirements**

- Please ensure that your manuscript meets PLOS ONE's style requirements, including those for file naming
  - Our manuscript was edited to fit with PLOS ONE’s style and file naming requirements
- Please include your full ethics statement in the ‘Methods’ section of your manuscript file.
  - Our ethics statement can now be found in the ‘Method’ section of our manuscript file.
- Please remove any funding-related text from the manuscript and let us know how you would like to update your Funding Statement
  - An amended funding statement can now be found in the cover letter for this revised submission
- We note that you have stated that you will provide repository information for your data at acceptance. Should your manuscript be accepted for publication, we will hold it until you provide the relevant accession numbers or DOIs necessary to access your data.
  - The information repository containing all data used in this study can be found in DNA Nexus and can be accessed via the following url: https://platform.dnanexus.com/projects/G6vJyPj0BYFv2KBJ4f55jkgf/data/
- Please include captions for your Supporting Information files at the end of your manuscript, and update any in-text citations to match accordingly.
  - Supporting information captions are now included at the end of the manuscript

**Review Comments**

- The STR loci were generated by GangSTR, however, the data validation is not performed as required from genomic sequencing data. At least the STR data should be confirmed by a different approach/tool for analyzing STR lengths and the selected samples should be sequenced by Sanger sequencing for the further validation.
  - Thank you for your response. All positions referred to have been now validated via Long Reads with subsets of those positions validated via Sanger Sequencing or gel electrophoresis. The wording in the *Validation* section and Supplemental Table S3 have been modified to reflect these additions.
- Thank you for asking me to review this manuscript. This indicates that expansion propensity varies depending on different haplotypes. I think that a study of ethnic diversity or of how a haplotype may correlate with a tendency to expansion is interesting. However we would require more detailed/systematic clinical data to see for example if these expansions may also explain some of the morbidity. But although the numbers of the cohorts mentioned in the manuscript are impressive, I am afraid that my expertise is not appropriate for making a judgment about the soundness of the Methods used.
  - Thank you for your comments. While we agree that additional clinical data would be valuable in examining the morbidity of many of these patients, we wanted to focus on the trends we noticed when identifying Short Tandem Repeats. We intend to pursue this information in subsequent studies.
